# Toupet Fundoplication With Re-sleeve for Hiatal Hernia Associated With Failed Sleeve Gastrectomy

**DOI:** 10.7759/cureus.96901

**Published:** 2025-11-15

**Authors:** Ahmed M Kandel, Ayman M Elwan, Mohamed Zeen, Mohamed Deyab, Abdorabih Alemam, Khaled Mohamed Salamh, Rasha Mohamed Motawea Ali, Ebrahim Abdelhalim, Masoud k El-syed Ibrahim, Mohamed Naroz, Atef A Hassan, Mohamed A Abdelaty

**Affiliations:** 1 General Surgery, Faculty of Medicine, Al-Azhar University, Cairo, EGY; 2 General Surgery, Faculty of Medicine for Girls, Al-Azhar University, Cairo, EGY; 3 Department of General Surgery, Faculty of Medicine, Horus University, Damietta, EGY; 4 Medicine and Surgery, Faculty of Medicine, Al-Azhar University, Cairo, EGY

**Keywords:** excess weight loss, gastroesophageal reflux disease, hiatal hernia, revisional bariatric surgery, toupet fundoplication

## Abstract

Background: Sleeve gastrectomy (SG) may induce or worsen gastroesophageal reflux disease (GERD), especially with coexisting hiatal hernia (HH). Evidence on combining Toupet fundoplication with re-sleeving is limited.

Objective: To assess the safety and effectiveness of Toupet fundoplication plus re-sleeve for reflux control and weight loss after failed SG with HH.

Methods: Prospective single-center series of 20 adults (30-60 years) with failed SG (≤50% excess weight loss at one year and/or BMI >35 kg/m²) and medically refractory GERD. Preoperative workup included endoscopy and contrast imaging. Surgery entailed complete hiatal dissection, posterior cruroplasty, 270° (Toupet) fundoplication using residual fundus with fixation to the crura, and re-sleeve over a 36-Fr bougie. The outcomes considered were: GERD resolution, complications, excess weight loss (EWL%) at three, six, and 18 months, and changes in comorbidities.

Results: The mean age of the patients was 42.8 years, 65% of the total patients were females, and the follow-up period was 18 months. HH was confirmed perioperatively in 55%; all had symptomatic GERD. The mean operative time was 100±22.6 minutes, and the hospital stay was 40±18.7 hours. No leaks, bleeding, reoperation, or mortality were observed; transient nausea in 40% resolved conservatively. GERD was resolved in 65% of the patients, 25% required proton pump inhibitors (PPIs) for three months, and 10% for six months, then as needed. No HH recurrence was observed. The excess weight loss percentage (EWL%) observed was as follows: 21% (three months), 42% (six months), 63.2% (18 months). Antihypertensives were discontinued. Diabetes remitted in 10% patients with dose reduction in one additional patient.

Conclusion: Toupet fundoplication with re-sleeve appears to be a safe and effective option for controlling GERD and promoting weight loss after failed SG with HH. Larger comparative studies are needed.

## Introduction

Obesity is associated with increased morbidity and mortality due to a higher risk of cardiovascular disease, osteoarthritis, diabetes, cancer, and gastroesophageal reflux disease (GERD) [1]. Up to 50% of patients with severe obesity report GERD symptoms [2]. Bariatric surgery is the only intervention with strong evidence for sustained, long-term weight loss [3]. GERD may arise de novo or worsen after sleeve gastrectomy (SG). Converting a compliant stomach into a narrow tubular conduit reduces compliance and raises intragastric pressure, particularly when the pylorus is closed. Disruption of anti-reflux anatomy, alteration of the angle of His and resection of sling fibers in the lower esophageal sphincter may further reduce sphincter pressure [4]. Sleeve configuration can also promote reflux: incisural narrowing, sleeve rotation, anatomic stenosis, residual fundus, and unrecognized hiatal hernia (HH) are reported contributors. Extensive antral resection may impair gastric emptying and increase reflux risk [4].

Failure after bariatric surgery is commonly defined as achieving less than 50% excess weight loss or regaining at least 50% of the weight initially lost [5,6]. After SG, approximately five to ten percent of patients require revisional surgery for persistent obesity, and recurrence of obesity-related comorbidities (e.g., hypertension and type 2 diabetes) is common with weight regain [7]. There is a growing consensus to actively search for HH during SG and repair it when identified [8]. An international consensus conference reported that 84% of bariatric surgeons routinely evaluate for HH and support repair when detected; multiple series describe concurrent HH repair at the time of SG [9]. Management options for weight regain after SG include redo SG, conversion to a malabsorptive procedure (e.g., gastric bypass), or augmentation of restriction (e.g., adjustable gastric band over sleeve) [10]. In patients undergoing SG with known HH, hiatal hernia repair (HHR) can be performed concurrently [11]. This study evaluates the safety and effectiveness of Toupet fundoplication combined with re-sleeve gastrectomy for patients with HH after failed SG, focusing on reflux control, weight loss, and perioperative outcomes.

## Materials and methods

Study design and setting

We conducted a prospective, single-center study at New Damietta University Hospital from April 2022 to April 2024. Consecutive adult patients with failed sleeve gastrectomy (SG) and symptomatic hiatal hernia (HH) scheduled for Toupet fundoplication with re-sleeve were enrolled.

Participants

Eligible patients were 30-60 years of age with failed SG, defined as ≤50% excess weight loss at one year and/or persistent body mass index (BMI) >35 kg/m² despite initial loss, and gastroesophageal reflux disease (GERD) that was refractory to medical therapy. Exclusion criteria included indications other than inadequate weight loss and GERD (e.g., excessive weight loss, medication nonadherence, significant nutritional problems, stenosis, or uncontrolled psychiatric disease), follow-up of less than two years after the index SG, and inadequate residual fundus precluding fundoplication.

Preoperative assessment

All patients underwent upper gastrointestinal endoscopy, a barium swallow, computed tomography (CT) gastric volumetry, and contrast-enhanced abdominal CT as clinically indicated.

Surgical technique

**Figure 1:**
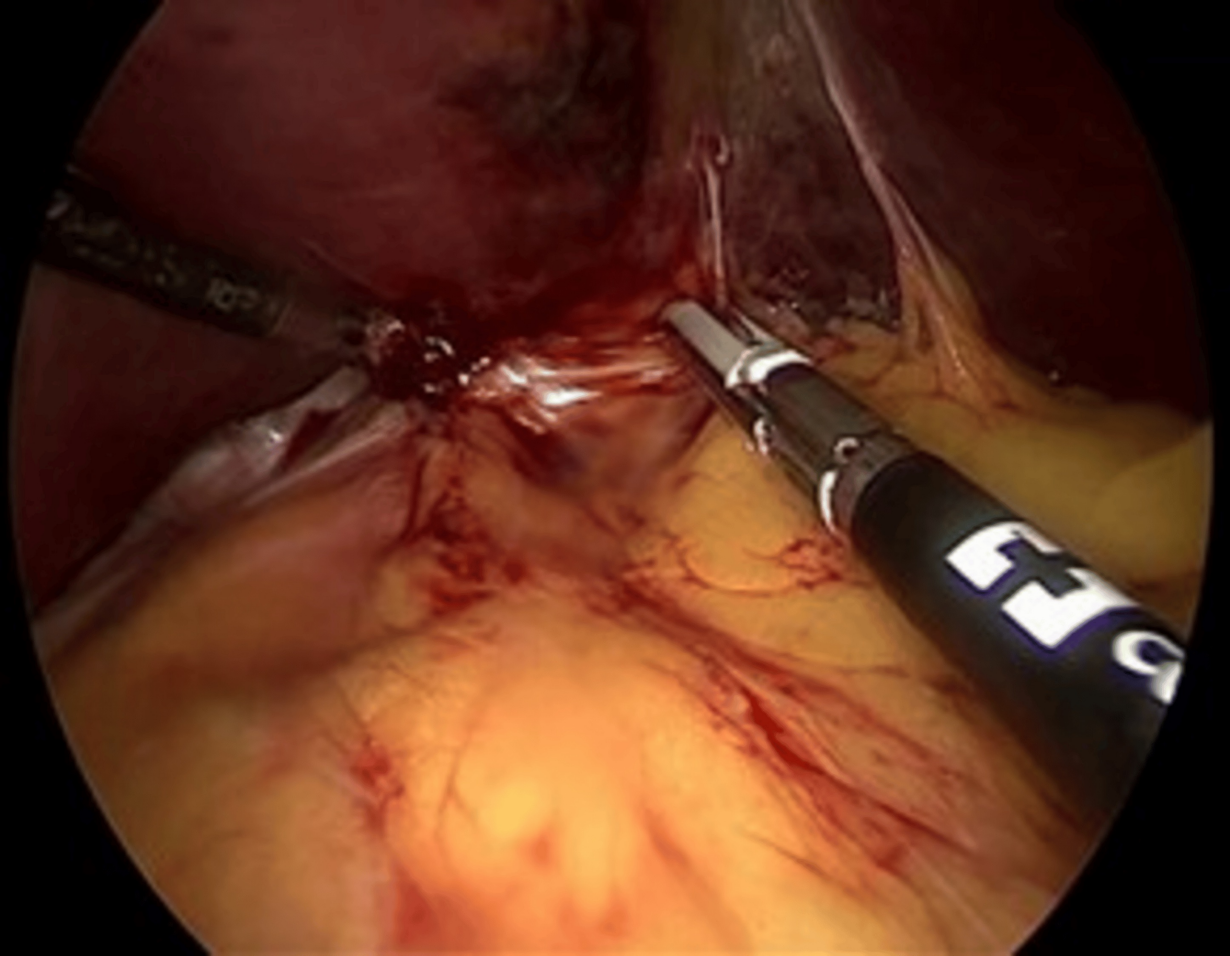
Dissection of the gastric pouch from the liver Dissection of the hepatogastric ligament and adhesions. The left hepatic lobe (large organ, top) is retracted, while surgical instruments (center) mobilize the proximal gastric pouch from its attachments

**Figure 2:**
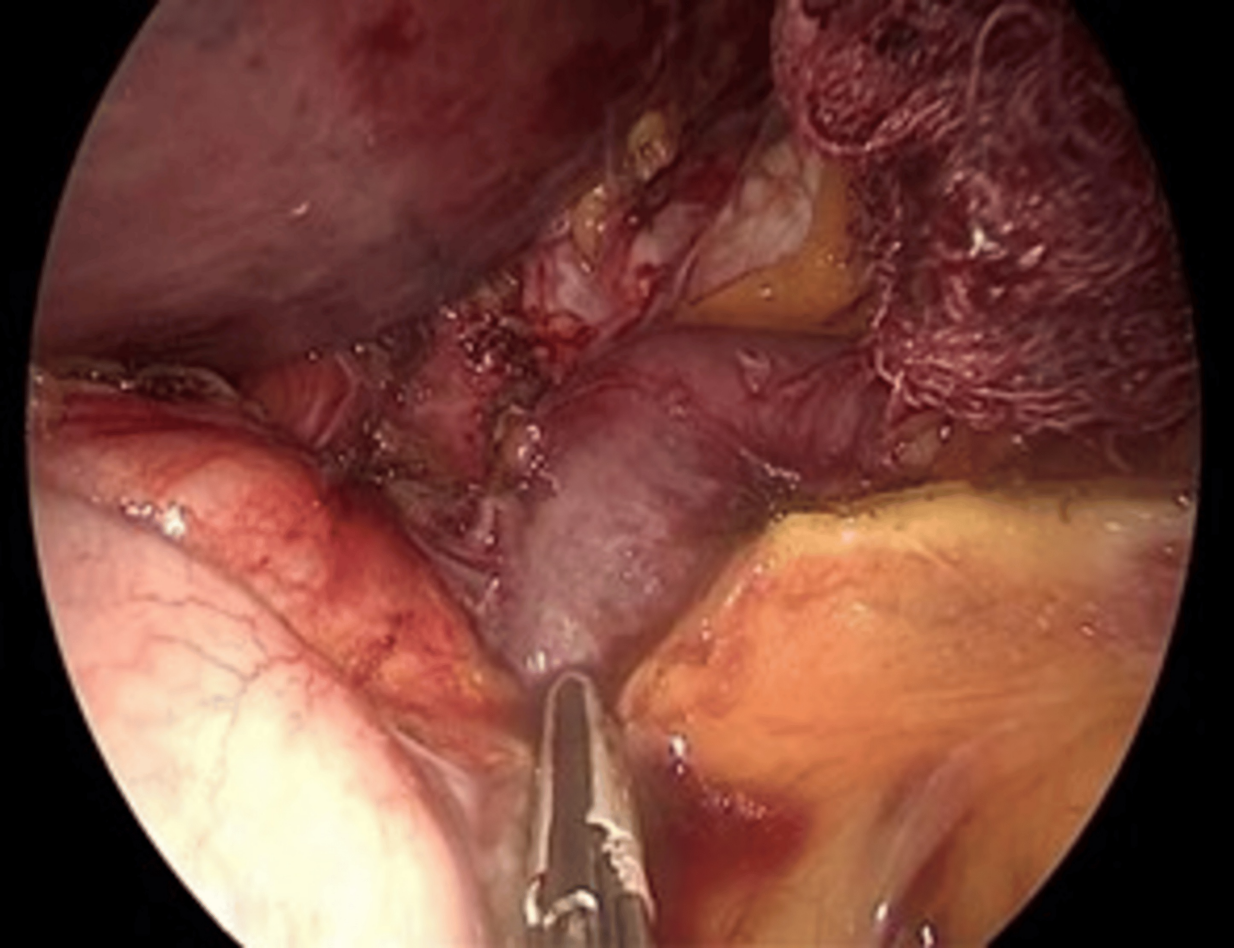
Complete fundus mobilization with exposure of the spleen The residual gastric fundus (tissue held by the instrument, bottom center) is freed from the short gastric vessels. The spleen is visible laterally (fibrous-looking organ, top right).

**Figure 3:**
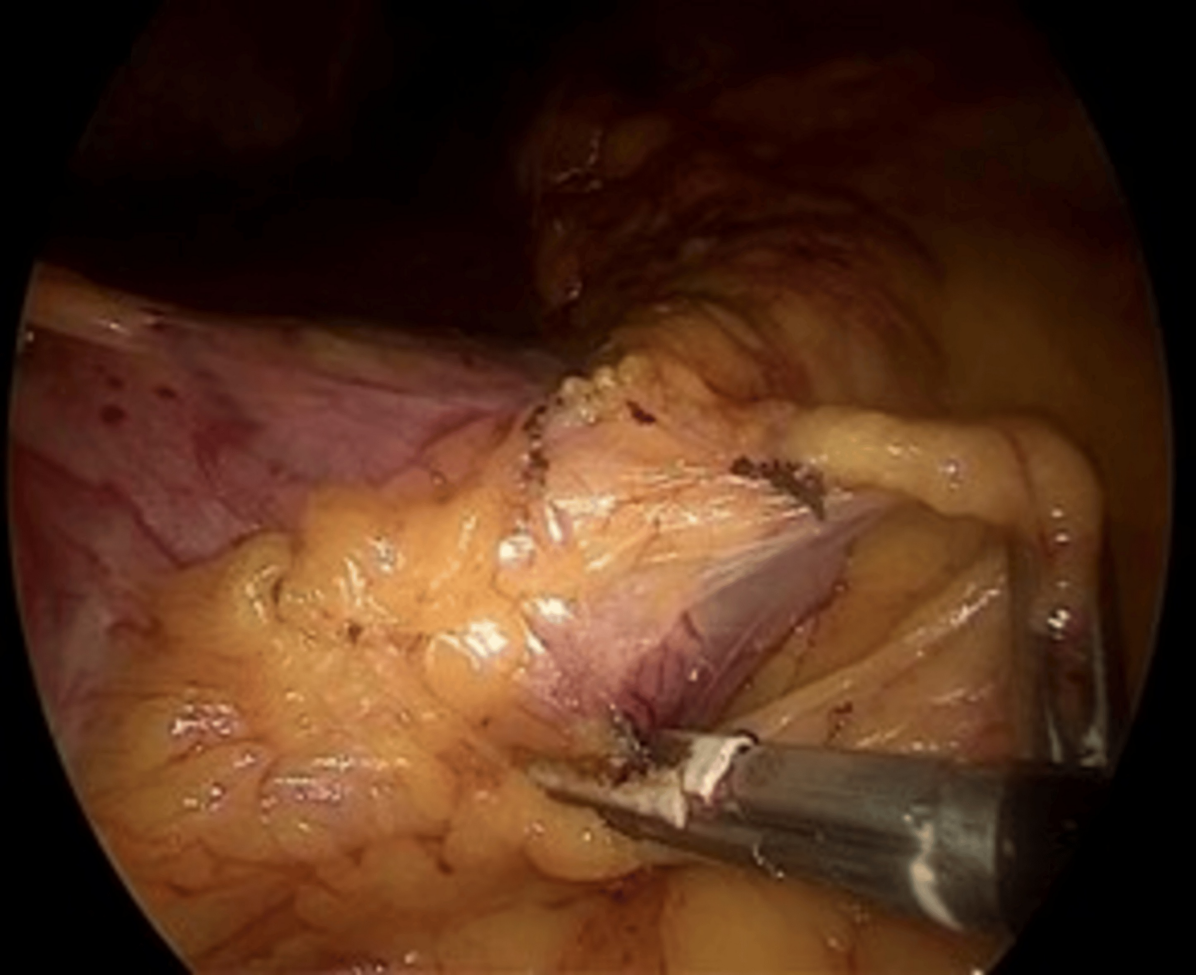
Division of retrogastric adhesions The energy device (instrument at bottom right) divides posterior attachments and retrogastric adhesions, mobilizing the gastric pouch from the posterior body wall.

**Figure 4:**
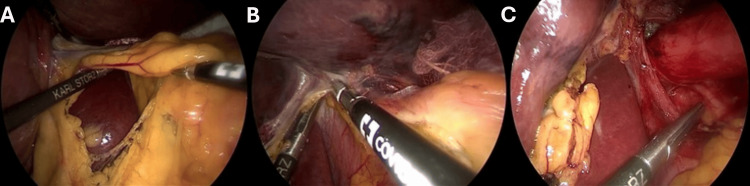
Hiatal dissection (composite) (A) An instrument divides the pars flaccida of the lesser omentum to access the right crus. (B) Dissection of the phrenoesophageal ligament to mobilize the distal esophagus (large muscular tube, right). (C) Identification and preservation of the posterior vagus nerve (white cord-like structure, center) running parallel to the esophagus.

**Figure 5:**
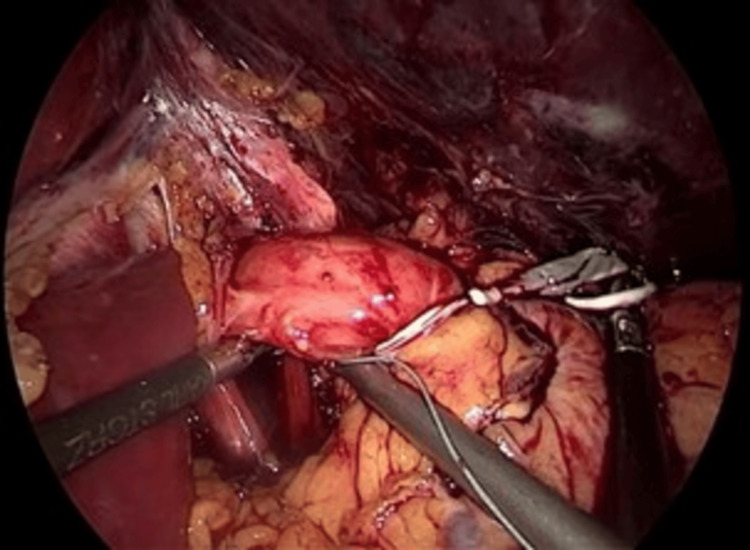
Posterior cruroplasty The right and left crura (muscular pillars behind the esophagus) are approximated with interrupted non-absorbable sutures. The distal esophagus (pale organ, center) is visible as the hiatal aperture is restored posteriorly.

**Figure 6:**
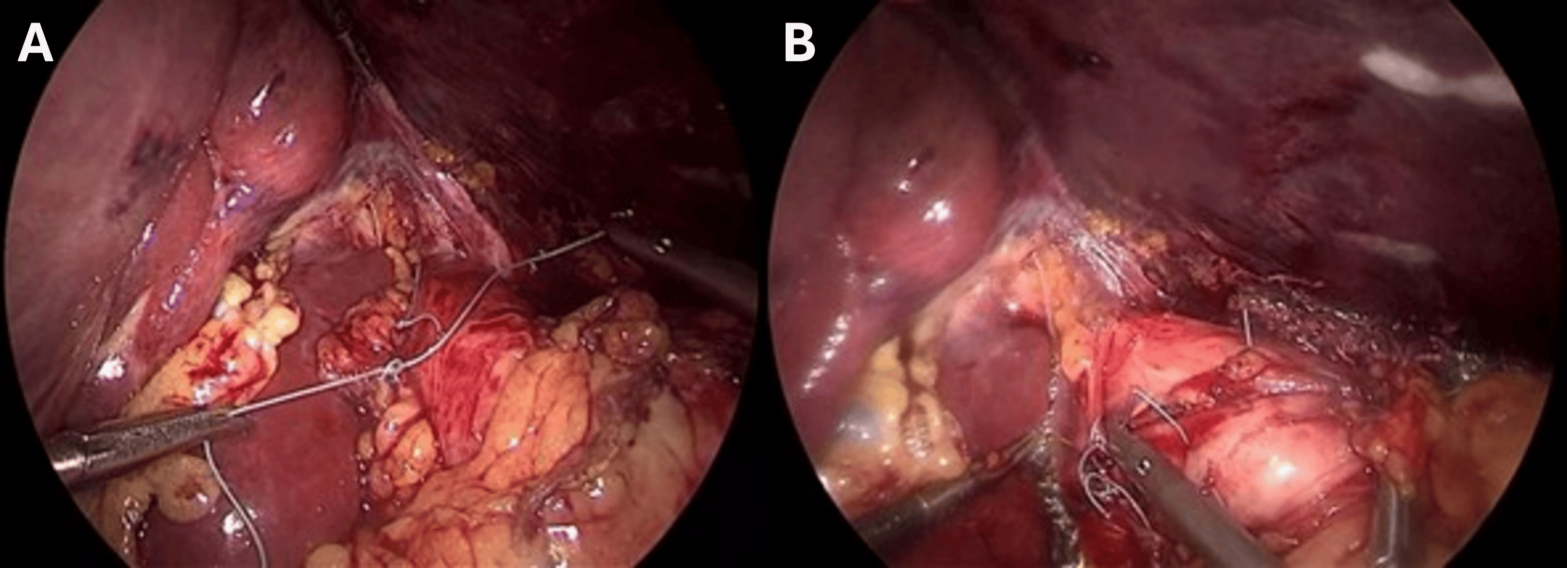
Toupet fundoplication (composite) (A) Construction of a 270° posterior wrap by passing the residual fundus (grasped by instrument, left) posterior to the esophagus (muscular tube, center-right). (B) Fixation of the completed fundic wrap to the adjacent right and left crura with sutures.

Under general anesthesia, patients received 2 g of a third-generation cephalosporin at the time of induction. Patients were positioned in reverse Trendelenburg with split legs. Five trocars were placed, and adhesiolysis was performed under direct vision using advanced bipolar energy. The gastric pouch was mobilized from the liver (Figure 1), the fundus was completely freed with exposure of the left crus (Figure 2), and retrogastric adhesions were divided until full mobilization of the pouch (Figure 3). The pars flaccida was opened to dissect the right crus while preserving the posterior Vagus nerve, and the phrenoesophageal ligament was divided. Circumferential esophageal mobilization allowed skeletonization of both crura, followed by posterior cruroplasty with two or three non-absorbable sutures (Figure 4). A 270° posterior Toupet fundoplication was fashioned using the residual fundus and anchored to the crura (Figures 5, 6).

Perioperative management

**Figure 7:**
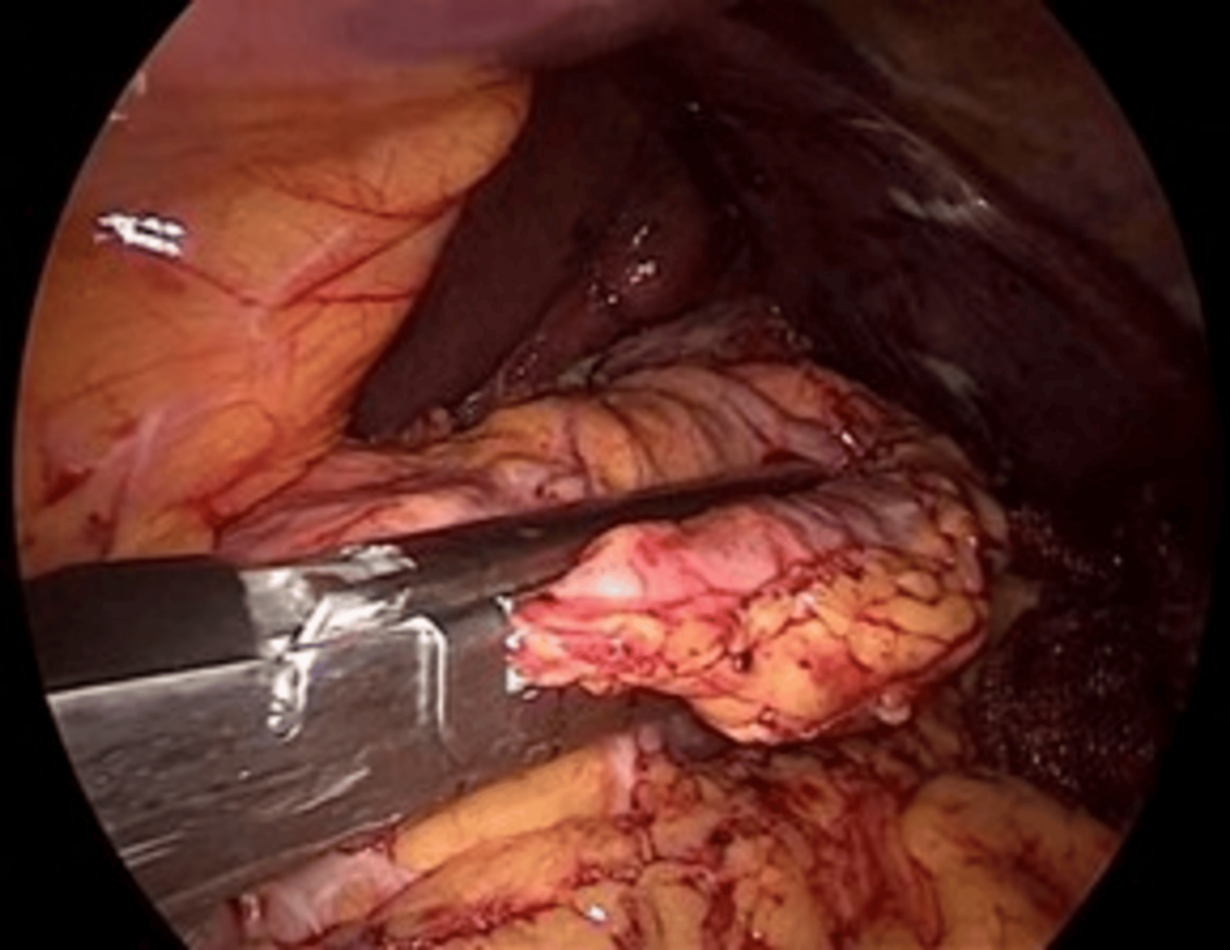
Re-sleeve over a 36-Fr orogastric tube Sequential stapling along the lesser curvature using a surgical stapler (large instrument, bottom left). The 36-Fr calibration tube (pink tube, center) is visible inside the gastric pouch to ensure proper sleeve sizing.

**Figure 8:**
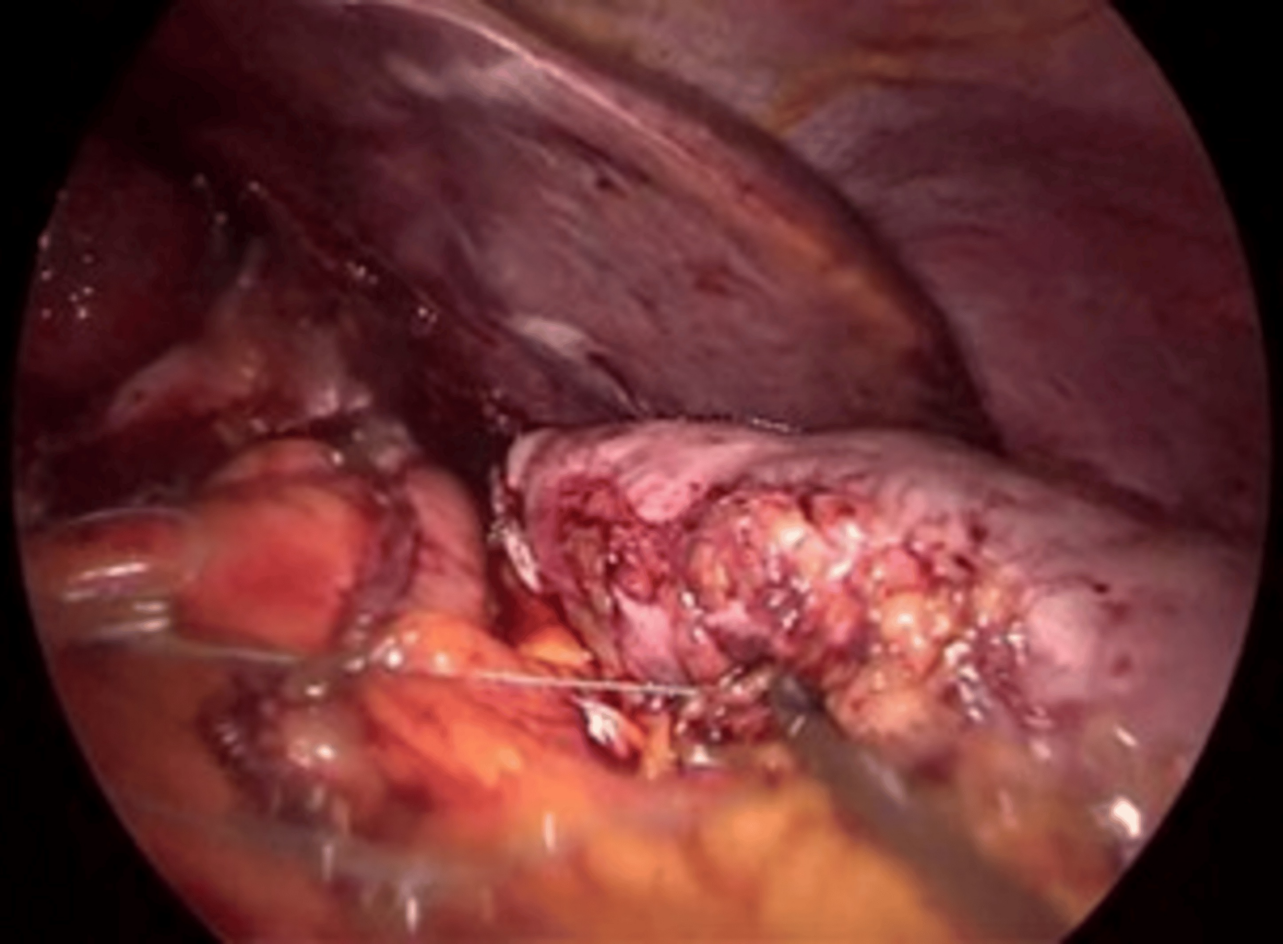
Gastropexy of the sleeve to the greater omentum The gastric sleeve staple line (center, running horizontally) is tacked with sutures (held by instrument, bottom right) to the greater omentum (fatty tissue, bottom) to minimize torsion.

Re-sleeve gastrectomy was then performed over a 36-Fr orogastric tube (Figure 7), and the gastric pouch was tacked to the greater omentum to minimize torsion and staple-line stress (Figure 8). A closed-suction drain was placed at the surgeon’s discretion, and port sites were closed after confirming hemostasis.

Standard thromboembolism prophylaxis, multimodal analgesia, and antiemetics were administered according to institutional protocols. An oral contrast study or selective imaging was obtained when clinically indicated. Diet was advanced in stages according to bariatric pathways.

Outcomes

The primary outcome was GERD control at follow-up, defined as complete symptom resolution without the use of proton pump inhibitors (PPIs) or improvement with reduced PPI requirements. Secondary outcomes included perioperative complications (leak, bleeding, reoperation, mortality), HH recurrence, excess weight loss (EWL%) at three, six, and 18 months, hospital length of stay, and changes in obesity-related comorbidities (hypertension and diabetes).

Follow-up and data collection

Patients were reviewed at three, six, and 18 months. Assessments included GERD symptoms and PPI use, weight and BMI to calculate EWL%, and evaluation of comorbidity status. Endoscopy or imaging was performed when clinically indicated. Data were prospectively recorded in a secure database.

Sample size rationale

This was an exploratory series of consecutive eligible patients during the defined study period; no formal power calculation was performed.

Statistical analysis

Continuous variables are reported as the mean ± standard deviation, and categorical variables as counts and percentages. Analyses were descriptive and conducted using SPSS software, version 30 (IBM Corp., Armonk, NY). A two-sided α of 0.05 was prespecified for any comparative analyses, although no hypothesis testing was planned.

Ethical consdierations

The study was approved by the Ethical Committee of Damietta Faculty of Medicine (DFM-IRB 00012367-24-11-003), and written informed consent was obtained from all participants in accordance with the Declaration of Helsinki.

## Results

Twenty patients underwent Toupet fundoplication with re-sleeve between April 2022 and April 2024 and completed follow-up at 18 months. The mean age was 42.8 ± 9.1 years; seven patients (35%) were male and 13 (65%) were female. Baseline characteristics and endoscopic grading are summarized in Table 1.

**Table 1:** Baseline characteristics Data are mean ± SD (range) or n (%). GERD: gastroesophageal reflux disease.

Variables	Value
Age, years, Mean ± SD (range)	42.8 ± 9.1 (30–60)
Male, n (%)	7 (35)
Female, n (%)	13 (65)
GERD symptoms, n (%)	20 (100)
Esophagitis grade (Los Angeles) — A, n (%)	3 (15)
Esophagitis grade (Los Angeles) - B, n (%)	8 (40)
Esophagitis grade (Los Angeles) - C, n (%)	7 (35)
Esophagitis grade (Los Angeles) - D, n (%)	2 (10)
Hiatal hernia (Hill grade) - II, n (%)	11 (55)
Hiatal hernia (Hill grade) - III, n (%)	5 (25)
Hiatal hernia (Hill grade)- IV, n (%)	3 (15)

Baseline and perioperative findings

All patients were symptomatic with gastroesophageal reflux disease (GERD) preoperatively. Esophagitis was graded according to the Los Angeles classification as follows: A in three patients (15%), B in eight (40%), C in seven (35%), and D in two (10%). Hiatal hernia was present in 11 patients (55%) based on intraoperative assessment (Hill grade II in eleven (55%), III in five (25%), and IV in three (15%)). The mean body mass index (BMI) was 43.3 ± 5.3 kg/m² (range 36-52). Hypertension and diabetes mellitus were present in five (25%) and three (15%) patients, respectively. The mean operative time was 100 ± 22.6 minutes (range 60-120), and the mean hospital stay was 40 ± 18.7 hours (range 24-72). Perioperative comorbidities and operative metrics are summarized in Table 2.

**Table 2:** Perioperative data Data are mean ± SD (range) or n (%). BMI: body mass index.

Variables	Value
Hypertension, n (%)	5 (25)
Diabetes mellitus, n (%)	3 (15)
BMI, kg/m², Mean ± SD (range)	43.3 ± 5.3 (36–52)
Operative time, minutes, Mean ± SD (range)	100 ± 22.6 (60–120)
Hospital stay, hours, Mean ± SD (range)	40 ± 18.7 (24–72)

Postoperative safety outcomes

There were no staple-line leaks, clinically significant postoperative bleeding events, reoperations, or deaths. Transient postoperative nausea occurred in eight patients (40%) and resolved with conservative management. No hiatal hernia recurrences were observed during the 18‑month follow-up interval.

Reflux control

At 18 months, GERD symptoms were resolved without proton pump inhibitors (PPIs) in 13 patients (65%). Five patients (25%) required PPIs for three months, and two patients (10%) required PPIs for six months, followed by as‑needed use.

Weight loss and comorbidity outcomes

Mean excess weight loss (EWL%) was 21 ± 8.4% at three months, 42 ± 9.6% at six months, and 63.2 ± 16.1% at 18 months. Antihypertensive therapy was discontinued in all previously hypertensive patients. Diabetes mellitus is remitted in two patients (10%), with dose reduction in one additional patient. EWL% milestones and comorbidity changes are shown in Table 3.

**Table 3:** Postoperative outcomes through 18 months PRN: as needed; PPI: proton pump inhibitor; EWL%: excess weight loss percentage.

Outcome (unit)	Value
Leak, n (%)	0 (0%)
Postoperative bleeding, n (%)	0 (0%)
Reoperation, n (%)	0 (0%)
Mortality, n (%)	0 (0%)
Nausea, n (%)	8 (40%)
Hiatal hernia recurrence, n (%)	0 (0%)
Hypertension on medication at last follow-up, n (%)	0 (0%)
Diabetes mellitus - remission, n (%)	2 (10%)
Diabetes mellitus - dose reduction, n (%)	1 (5%)
GERD status - resolved, n (%)	13 (65%)
GERD status - PPI three months, n (%)	5 (25%)
GERD status - PPI six months then PRN, n(%)	2 (10%)
EWL% - three months, mean ± SD	21 ± 8.4
EWL% - six months, mean ± SD	42 ± 9.6
EWL% - 18 months, mean ± SD	63.2 ± 16.1

## Discussion

Summary of principal findings

In this prospective series of 20 patients with failed sleeve gastrectomy (SG) and symptomatic hiatal hernia (HH), Toupet fundoplication combined with re-sleeve was associated with favorable safety and clinical efficacy at 18 months. There were no leaks, postoperative bleeding events, reoperations, or deaths. Reflux symptoms resolved without proton pump inhibitors (PPIs) in 65% of patients, with additional improvement among those requiring short-term PPIs. Mean excess weight loss increased over time (21% percent at three months, 42% at six months, and 63% percent at 18 months), with improvement in obesity-related comorbidities and no HH recurrences observed.

Context within literature

Weight-loss “failure” after bariatric surgery encompasses insufficient excess weight loss and clinically meaningful weight regain, each of which carries metabolic and quality-of-life consequences [12,13]. After SG, gradual weight regain and de novo or progressive gastroesophageal reflux disease (GERD) are well described [14]. Revisional strategies for GERD and inadequate weight loss include re-sleeve and conversion procedures. Roux-en-Y gastric bypass (RYGB) is often favored when reflux predominates, but the choice should be individualized, given the trade-offs of a malabsorptive reconstruction [15,16]. Traditional teaching holds that fundoplication is generally not feasible after SG due to limited residual fundus; however, selected patients may retain sufficient fundic tissue to permit a partial (270°) wrap [17].

Appropriate HH repair is central to reflux control in this setting. Key principles include mediastinal esophageal mobilization, reduction of the hernia sac (when present), and posterior cruroplasty [18]. Evidence supporting attention to the hiatus at the time of SG continues to accumulate; reports of SG with concurrent HH repair describe symptom improvement and reduced PPI use compared with SG alone [19,20]. Persistent or refractory GERD after SG is a frequent indication for revisional surgery, often conversion to RYGB with good reflux control, but at the cost of greater operative complexity and potential nutritional consequences [21]. In our series, we combined posterior cruroplasty with Toupet fundoplication, then reinforced the restriction via re-sleeve, offering reflux control without conversion to a malabsorptive procedure.

Technical considerations

Our approach emphasized complete hiatal dissection, tension-free posterior cruroplasty, and a 270° posterior fundic wrap fashioned from the residual fundus and anchored to the crura. This respects anti-reflux mechanics while acknowledging an altered gastric anatomy after SG. We also performed gastropexy of the sleeve to the greater omentum to reduce torsion and staple-line stress. Although mesh can reduce mid-term recurrence in large or complex hiatal defects, guideline statements and contemporary reviews underscore ongoing debate regarding mesh type, configuration, and fixation, and highlight rare but serious mesh-related complications [22,23]. Given these concerns and the absence of large paraesophageal defects in our cohort, we favored suture repair without the use of a mesh.

Alternative revisional options

Conversion to RYGB remains a benchmark for reflux control after SG, with robust anti-reflux physiology and additional weight-loss potential [16,20]. However, this entails different risk and nutritional profiles compared with a restrictive approach. Re-sleeve alone may improve weight control but is less reliable for reflux when an HH persists or anti-reflux mechanisms are not restored [15,16]. Our results suggest that a carefully executed Toupet fundoplication with cruroplasty, plus re-sleeve, may bridge this gap in selected patients who retain adequate fundus and prefer to avoid malabsorption.

Limitations

This study is limited by its single-center design, small sample size, absence of a control group, and reliance primarily on symptom-based GERD assessment without routine pH impedance or high-resolution manometry. Follow-up was limited to eighteen months, which may not capture late hernia recurrence, wrap failure, or long-term weight trajectories. Selection bias is possible given that adequate residual fundus was required for fundoplication.

Implications and future directions

For patients with failed SG and HH who retain a sufficient fundus, Toupet fundoplication with cruroplasty plus re-sleeve may provide reflux control and clinically meaningful weight loss while avoiding the nutritional trade-offs associated with conversion to RYGB. Future work should include prospective comparative studies (e.g., propensity-matched cohorts or randomized trials) against RYGB and re-sleeve alone, incorporating standardized objective reflux testing and disease-specific quality-of-life measures, and extend follow-up beyond thirty-six months to evaluate durability.

## Conclusions

Toupet fundoplication with posterior cruroplasty combined with re-sleeve was safe and feasible in this series, yielding meaningful reflux control and progressive excess weight loss at 18 months without hiatal hernia recurrence. For carefully selected patients with failed sleeve gastrectomy and hiatal hernia who retain adequate fundus, this approach may offer a non-malabsorptive alternative to conversion procedures. Larger comparative studies with objective reflux assessment and longer follow-up are warranted.
